# Understanding Food Choices Among University Students: Dietary Identity, Decision-Making Motives, and Contextual Influences

**DOI:** 10.3390/nu18020228

**Published:** 2026-01-12

**Authors:** Ali Aboueldahab, Maria Elide Vanutelli, Marco D’Addario, Patrizia Steca

**Affiliations:** Department of Psychology, University of Milano-Bicocca, 20126 Milan, Italy; maria.vanutelli@unimib.it (M.E.V.); marco.daddario@unimib.it (M.D.); patrizia.steca@unimib.it (P.S.)

**Keywords:** university students, eating behavior, food choice, canteen, dietary identity, nudging

## Abstract

**Background:** Dietary habits established during young adulthood have long-term implications for health, and food choices among university students are strongly shaped by contextual factors. Institutional eating environments represent a relevant setting for promoting healthier dietary behaviors, yet limited evidence integrates students’ engagement with these settings, their food consumption patterns across contexts, and the individual decision-making processes underlying food choice. **Methods:** This cross-sectional study analyzed survey data from 1519 students enrolled at a large Italian university. Measures included sociodemographic characteristics, self-identified dietary style, engagement with the university canteen, consumption frequency of selected food categories across institutional and non-institutional contexts, and category-specific food-choice motivations. Data were analyzed using descriptive analyses, Borda count rankings, paired comparisons, and multiple linear regression models. **Results:** Clear contextual differences in food consumption emerged across all food categories, with consistently lower consumption frequencies within the university canteen compared to outside settings (all *p* < 0.001). The largest contextual gap was observed for fruit consumption (d = 0.94), with similarly pronounced differences for plant-based foods. Taste was the most salient decision-making factor across food categories (overall M ≈ 4.4), while health-related motives were more prominent for healthier foods and gratification for desserts. Across contexts, self-identified dietary style was the most consistent predictor of food consumption, explaining substantial variance for animal-based protein consumption (R^2^ = 0.293 in the canteen; R^2^ = 0.353 outside), whereas age and gender showed smaller, food-specific associations. **Conclusions:** The findings highlight institutional eating settings as distinct food environments in which individual dietary preferences are only partially expressed. Effective strategies to promote healthier eating among university students should move beyond generic approaches and integrate interventions targeting service-related engagement, category-specific choice architecture, and students’ dietary identities.

## 1. Introduction

Dietary habits established during young adulthood play a crucial role in shaping both current and long-term health and well-being. Suboptimal dietary patterns—such as low consumption of fruits and vegetables, high intake of refined grains and energy-dense foods, and irregular eating routines—are consistently associated with increased risks of obesity, type 2 diabetes, cardiovascular disease, and poorer mental health outcomes across the lifespan [1,2,3,4]. Because eating behaviors developed early in adulthood often persist over time, this life stage represents a particularly sensitive window for the consolidation of dietary habits with lasting health implications [5,6,7].

Young adulthood is also a critical developmental phase for the formation of stable behavioral and identity-related patterns. A growing body of research suggests that the university period constitutes a key context in which enduring habits are formed, not only in relation to general lifestyles but also to health-related behaviors, including diet [5,7,8]. University students are often exposed to a combination of increased autonomy, academic demands, time constraints, and social influences, all of which contribute to the stabilization of everyday routines and decision-making patterns. As a result, dietary behaviors adopted during this phase may become deeply embedded and resistant to change later in life [4].

Within this context, universities represent a highly influential food environment. During academic periods, students spend a substantial proportion of their daily time on campus and frequently rely on institutional food services and nearby commercial outlets for their meals. Research on campus food environments shows that students’ eating behaviors are shaped not only by individual preferences but also by contextual and structural factors such as food availability, pricing, convenience, time pressure, and perceived quality [9,10,11]. Among these environments, university canteens play a particularly strategic role. Their standardized menus, regulated prices, and stable food offerings make them key settings for population-level interventions aimed at improving dietary quality among students [12,13].

Consistent with this view, a growing body of evidence indicates that behavioral interventions implemented in canteen settings—such as nudging strategies, changes in food architecture, improved presentation of healthier options, or informational cues—can effectively influence food choices, often with effects that extend beyond the immediate decision context [14,15,16,17,18]. These findings highlight the potential of university canteens as leverage points for promoting healthier eating behaviors at scale. However, the effectiveness of such interventions depends not only on the nutritional quality of the food offered but also on students’ actual engagement with the canteen service.

Indeed, focusing exclusively on the canteen as a physical setting provides only a partial understanding of students’ eating behaviors. Food choices are inherently context-dependent, and decisions made within institutional environments may differ substantially from those made in more autonomous contexts, such as eating at home or at commercial food outlets [11,19]. Moreover, students’ decisions to use or avoid university canteens are influenced by multiple factors beyond food composition, including perceived quality, cost, waiting times, convenience, and the social and physical dining environment [20,21].

When canteens are perceived as inconvenient or of low value, students may shift toward alternative food sources that are often less nutritious [22,23]. Examining both reasons for use and non-use is therefore essential to accurately assess students’ exposure to the campus food environment and the realistic reach of canteen-based health interventions [24].

At the individual level, dietary behaviors vary systematically according to sociodemographic characteristics and stable lifestyle orientations. Age, gender, and self-identified dietary styles (e.g., omnivorous, flexitarian, vegetarian, vegan) have been consistently associated with differences in food preferences, motivations, and consumption patterns among young adults [12,25,26,27]. In addition, research on food choice increasingly emphasizes that decision-making processes are highly food specific. Factors such as healthiness, taste, gratification, digestibility, cost, appearance, habit, and ethical considerations do not contribute uniformly across food categories but vary depending on the type of food being selected [12,28,29]. Ignoring these category-specific dynamics risks obscuring key psychological mechanisms underlying dietary behavior.

Despite extensive evidence on the importance of dietary habits, the role of the university as a formative context, and the effectiveness of canteen-based interventions, relatively few studies have attempted to integrate these perspectives. Existing research has often focused on food availability or nutritional quality alone, with limited attention to students’ motivations and identities as drivers of food choice across contexts [30,31]. There is a lack of research simultaneously examining eating behaviors inside and outside the university context, individual lifestyle characteristics, engagement with the canteen service, and food-specific decision-making processes within a single analytical framework.

Against this background, the present study aims to provide an integrated analysis of eating behaviors among students at a large Italian university, with a particular focus on the role of the university canteen. Using a cross-sectional design, the study seeks to: (i) describe students’ habitual dietary styles and food consumption patterns outside the university context; (ii) examine consumption frequencies and food-choice motivations for specific food categories within the canteen; and (iii) explore the factors associated with the use and non-use of the canteen service. By connecting individual characteristics, contextual factors, and category-specific decision-making processes, the study aims to generate actionable insights to inform the development of targeted, context-sensitive interventions to promote healthier eating behaviors in university settings.

## 2. Methods

### 2.1. Participants and Setting

This cross-sectional study was conducted using an anonymous online survey administered to university students. The survey was developed and distributed using Qualtrics (Qualtrics, Provo, UT, USA). Students could access the questionnaire by scanning QR codes displayed on screens located in common areas and university facilities during the academic period. The final sample consisted of 1519 university students. Most participants were aged between 18 and 30 years (95.1%), and 68.5% identified as women. Students were enrolled in undergraduate, master’s, and doctoral programs across a wide range of academic disciplines. Participation was voluntary, and no financial or academic incentives were provided. Eligibility criteria included being at least 18 years old, being enrolled as a student at the University of Milano-Bicocca at the time of survey completion and providing informed consent. Missing data were handled on an analysis-specific basis. Data were collected between June 2024 and April 2025.

The study was conducted in accordance with the Declaration of Helsinki, complied with the European General Data Protection Regulation (GDPR), and evaluated by the local commission for minimal-risk studies of the Psychology Department of University of Milano-Bicocca (Protocol no. RM-2024-814). All participants provided informed consent electronically, and all data were collected and stored in anonymized form.

### 2.2. Questionnaire Design

The questionnaire was developed as a non-standardized survey aimed at capturing food-related behaviors and dietary choices among university students. The item pool was constructed by a multidisciplinary research team with expertise in psychology and nutrition sciences, based on the study objectives and by adapting core items from the previous “Mangiare in Bicocca” survey to ensure continuity and comparability within the same institutional context [24].

Items assessing self-identified dietary style (e.g., omnivorous, flexitarian, pescatarian, vegetarian, vegan) were informed by prior research on reduced-meat dietary identities and flexitarianism. Particular attention was paid to capturing differences in food-choice motivations across food categories, in line with evidence that food-related decision-making processes are category- and context-specific rather than uniform across choices [26,27]. Prior to data collection, the questionnaire was pilot tested on a small convenience sample of approximately 30 university students to assess clarity, comprehensibility, and completion time. Minor revisions to item wording, response options, and question flow were implemented based on participant feedback and expert judgment.

The final questionnaire included the following sections: (i) sociodemographic characteristics; (ii) self-identified dietary style; (iii) use and frequency of use of the university canteen; (iv) perceived importance of factors associated with using or avoiding the canteen; (v) frequency of consumption of selected food categories within the canteen and the perceived importance of food-choice motives for each category; (vi) frequency of consumption of the same food categories outside the university context.

Sociodemographic variables included age range, gender, and university role. Food consumption was assessed across six food categories: animal-based proteins, plant-based protein dishes, white bread, whole-grain bread, desserts, and fruit. These categories were selected based on prior literature highlighting substantial differences in both perceived healthiness and objective nutritional profiles across food types, as well as in the values and meanings individuals associate with them in food-related decision-making processes [28,29,32,33]. To facilitate interpretation, participants were provided with brief clarifications for each food category. Animal- and plant-based protein categories were accompanied by illustrative examples to distinguish main dishes based on animal versus plant sources. In line with typical Italian university canteen practices, bread was considered exclusively as a side dish accompanying main meals, with a distinction between white (refined) and whole-grain options. Fruit and desserts were also defined according to standard canteen offerings, with fruit typically available as whole fruit or fruit salads, and desserts including prepared sweet items such as cakes or puddings.

In particular, previous studies have shown that foods such as plant-based dishes, whole grains, and fruit are typically perceived as healthier and more aligned with long-term health goals, whereas animal-based proteins, refined grains, and desserts are more strongly associated with hedonic value, gratification, and immediate sensory rewards [32,33]. These contrasts make such categories especially informative for examining category-specific decision-making mechanisms.

An additional criterion guiding the selection of these food categories was their widespread availability both within institutional eating contexts and outside the university setting. This allowed for a meaningful comparison of consumption patterns across contexts while holding food category constant, thereby supporting the examination of contextual influences on students’ dietary choices.

For each category, participants reported frequency of consumption both within the canteen and outside the university context using 5-point frequency scales. For canteen-specific choices, participants also rated the importance of multiple food-choice motives (healthiness, taste, digestibility, gratification, cost, appearance, habit, and ethical considerations) on 5-point Likert-type scales.

The survey was administered online via Qualtrics. Conditional skip logic was implemented so that canteen-specific sections were displayed only to participants reporting prior canteen use. An overview of questionnaire sections and assessed constructs is provided in Table 1.

### 2.3. Statistical Analysis

All statistical analyses were conducted using IBM SPSS Statistics v. 29.0 (IBM Corp., Armonk, NY, USA). Descriptive statistics were computed to summarize sample characteristics and main study variables. Categorical variables were reported as frequencies and percentages, while continuous variables were summarized using means and standard deviations.

Food consumption patterns and lunch-related behaviors were examined separately for meals consumed within the university canteen and outside the university context. Mean frequency scores were calculated for each food category, and differences between food categories and contexts were examined using paired-sample *t*-tests or repeated-measures comparisons, as appropriate.

To assess the relative importance of factors influencing the use and non-use of the university canteen, ranking data were analyzed using a Borda count procedure. Inverse scores were assigned to ranked positions, summed across participants, and normalized to obtain weighted mean scores representing the relative salience of each factor.

Group comparisons were conducted using independent-sample *t*-tests or one-way analyses of variance (ANOVA), with Bonferroni or Games–Howell post hoc corrections applied as appropriate. Associations between continuous variables were examined using Pearson correlation coefficients. Multiple linear regression analyses were performed to examine predictors of food consumption frequencies within and outside the canteen. Predictor variables included age, gender, self-identified dietary style, and selected behavioral variables where available. Regression assumptions were checked prior to interpretation. Results are reported as unstandardized coefficients (B), standardized coefficients (β), and explained variance (R^2^).

All statistical tests were two-tailed, and a *p*-value ≤ 0.05 was considered statistically significant.

## 3. Results

Results are presented following a progressive logic. First, there is a description of the sample characteristics and lunch arrangements during the academic period.

Second, patterns of university canteen use and the main reasons for use and non-use are reported.

Third, consumption frequencies of selected food categories inside and outside the canteen are compared.

Finally, food-choice decision-making factors and individual predictors of food consumption across contexts are examined.

### 3.1. Sample Characteristics

The analyzed sample consisted of 1519 university students, the majority of whom were aged between 18 and 30 years (95.1%). Most participants identified as women (68.5%), followed by men (28.9%), non-binary/third gender (1.4%), or preferred not to disclose their gender (1.2%). Regarding self-identified dietary style, most respondents reported an omnivorous diet (77.4%), followed by flexitarian (10.6%), vegetarian (5.7%), vegan (3.1%), and pescatarian (2.0%) patterns. Overall, 75.7% of participants reported having used the university canteen at least once. An overview of the sample characteristics is provided in Table 2. 

### 3.2. University Canteen Use

Overall, 75.7% of participants (N = 1117) reported having used the university canteen at least once, whereas 24.3% (N = 359) reported never using the service.

Among canteen users, the distribution of use frequency was highly variable: 22.8% reported using the canteen almost never, while 25.4% reported using it almost always.

The mean frequency of canteen use was 3.11 (SD = 1.50) on a 5-point scale, indicating a moderate overall level of utilization. Full frequency distributions are reported in Table 3.

### 3.3. Reasons for Using and Not Using the University Canteen

To better characterize students’ food-related decision-making, the analysis also examined the factors underlying students’ decisions to use or avoid the university canteen. This approach allows consideration of contextual and service-related factors that shape students’ exposure to institutional food environments, complementing the analysis of food choice patterns presented in the following sections. The factors included in this analysis were selected based on prior research conducted in the same university context [24].

#### 3.3.1. Reasons for Use

A Borda count analysis was conducted to assess the relative importance of factors influencing canteen use. Cost emerged as the most influential factor (mean Borda = 4.69), accounting for 22.4% of the total decision weight. In the Italian university context, canteen services are typically subsidized and often linked to students’ income brackets or scholarship status, resulting in meal prices that are substantially lower than those of surrounding commercial food outlets. Food quality (mean = 4.07; 19.4%) and distance from usual university locations (mean = 3.96; 18.9%) ranked second and third, respectively. Together, these three factors accounted for approximately 60% of the overall decision weight. Service time and the presence of friends or colleagues showed intermediate relevance, whereas habit ranked last. Detailed results are reported in Table 4.

#### 3.3.2. Reasons for Non-Use

For non-use of the canteen, service time (mean Borda = 3.44; 24.1%) and food quality (mean = 3.37; 23.8%) emerged as the most relevant deterrents, with very similar weights. Cost and canteen environment showed intermediate importance, whereas distance from usual locations ranked lowest. Full results of the Borda analysis for non-use are reported in Table 5.

### 3.4. Consumption of Selected Food Categories

Understanding food consumption patterns across different contexts is essential to capture how environmental settings shape students’ dietary behaviors. Comparing the frequency of consumption of selected food categories within institutional eating contexts and outside the university allows assessment of whether and how food choices vary as a function of context, beyond individual dietary preferences.

#### 3.4.1. Consumption in the Canteen

Within the university canteen, animal-based proteins showed the highest consumption frequency (M = 3.49, SD = 1.19), followed by plant-based proteins (M = 3.15, SD = 1.16). Bread products and desserts were consumed less frequently, with particularly low consumption of whole-grain bread (M = 2.11, SD = 1.28), which 47.0% of respondents reported never consuming in the canteen. Fruit consumption was similarly low (M = 2.11, SD = 1.30).

Descriptive statistics for all food categories are reported in Table 6.

#### 3.4.2. Consumption Outside the Canteen

Outside the canteen, consumption frequencies were higher for all food categories. Animal-based proteins (M = 3.74, SD = 1.01) and plant-based proteins (M = 3.64, SD = 0.97) showed high consumption levels, as did fruit (M = 3.69, SD = 1.14). Desserts and bread products were consumed with moderate frequency.

Full descriptive statistics are reported in Table 7.

#### 3.4.3. Comparison Between Contexts

Paired-samples *t*-tests indicated that consumption frequencies were significantly higher outside the canteen than within the canteen for all food categories (all *p* < 0.001). Effect sizes ranged from small for white bread (d = 0.16) and animal-based proteins (d = 0.27) to moderate for desserts (d = 0.53) and large for fruit consumption (d = 0.94), indicating substantial contextual differences in eating behavior.

Detailed test statistics are reported in Table 8.

### 3.5. Factors Influencing Food Choice Within the Canteen

Across all food categories, taste consistently emerged as the most important decision-making factor (overall M ≈ 4.4). Perceived healthiness was particularly salient for plant-based proteins (M = 4.14), whole-grain bread (M = 3.99), and fruit (M = 4.42), whereas it was rated as less important for desserts (M = 3.09). Gratification was especially relevant for desserts (M = 4.38), while habit and ethical considerations were consistently rated as the least important factors.

Overall, decision-making profiles differed systematically across food categories. Detailed descriptive statistics for each category are reported in Table 9.

### 3.6. Comparison of Decision-Making Factors for Healthier vs. Less Healthy Foods in the Canteen

Comparative analyses showed that healthiness was rated as substantially more important for healthier foods than for less healthy foods (M = 4.15 vs. 3.60; d = 0.64). Digestibility and ethical considerations also favored healthier foods, whereas gratification and cost were rated as slightly more important for less healthy foods. Taste showed no significant difference between healthier and less healthy food categories (*p* = 0.461).

Full results are reported in Table 10.

### 3.7. Predictors of Food Consumption in the University Canteen

To examine whether individual characteristics were associated with food consumption patterns within the university canteen, a series of multiple linear regression analyses was conducted. For each food category, consumption frequency in the canteen was entered as the dependent variable, while age, gender, and self-identified dietary style were included simultaneously as predictors using the enter method. Model summaries and regression coefficients are reported in Table 11.

Across food categories, self-identified dietary style emerged as the most consistent and influential predictor of consumption patterns. A stronger orientation toward dietary styles characterized by reduced or absent consumption of animal products was associated with lower consumption of animal-based proteins (β = −0.50, *p* < 0.001) and desserts (β = −0.16, *p* < 0.001), and with higher consumption of plant-based proteins (β = 0.34, *p* < 0.001), whole-grain bread (β = 0.14, *p* < 0.001), and fruit (β = 0.09, *p* = 0.006). These effects remained robust across models and showed the largest standardized coefficients among all predictors.

Age showed smaller but statistically significant associations for selected food categories. Higher age was associated with lower consumption of animal-based proteins, white bread, and desserts, and with higher consumption of whole-grain bread (all *p* ≤ 0.01).

Gender was significantly associated only with animal-based protein consumption and white bread intake, while no significant gender differences emerged for plant-based proteins, desserts, or fruit.

Model fit varied substantially across food categories. The model predicting animal-based protein consumption explained a substantial proportion of variance (R^2^ = 0.293), whereas the model for plant-based proteins showed more modest explanatory power (R^2^ = 0.122). Models predicting bread products, desserts, and fruit explained limited variance (R^2^ range = 0.008–0.045), indicating weaker associations with the individual characteristics considered.

### 3.8. Predictors of Food Consumption Outside the University Canteen

Across food categories, self-identified dietary style again emerged as the most consistent and influential predictor of food consumption. A stronger orientation toward dietary styles characterized by reduced or absent consumption of animal products was associated with lower consumption of animal-based proteins (β = −0.57, *p* < 0.001) and white bread (β = −0.11, *p* < 0.001), and with higher consumption of plant-based proteins (β = 0.36, *p* < 0.001), whole-grain bread (β = 0.16, *p* < 0.001), and fruit (β = 0.08, *p* = 0.009). Dietary style was not significantly associated with dessert consumption.

Age showed small but statistically significant associations for selected food categories. Higher age was associated with lower consumption of animal-based proteins, white bread, and desserts (all *p* < 0.001), whereas no significant associations emerged for plant-based proteins, whole-grain bread, or fruit.

Gender showed food-specific associations, being significantly related to the consumption of animal-based proteins, plant-based proteins, white bread, and desserts, while no significant gender differences emerged for whole-grain bread or fruit.

Model fit varied across food categories. The model predicting animal-based protein consumption explained a substantial proportion of variance (R^2^ = 0.353), whereas the model for plant-based proteins showed moderate explanatory power (R^2^ = 0.138). Models predicting bread products, desserts, and fruit explained limited variance (R^2^ range = 0.009–0.033), indicating weaker associations with the individual characteristics considered.

A summary of significant predictors across food categories and contexts is provided in Table 12.

## 4. Discussion

This study provides an integrated, multi-level analysis of university students’ eating behaviors, with a specific focus on the role of the institutional canteen. Grounded in a socio-ecological perspective that emphasizes the interplay between individual characteristics, environmental contexts, and specific decision-making processes [24,34], the research sought to bridge students’ general dietary identities, their patterns of canteen engagement, and the nuanced motivations underlying their food selections within that setting. By examining these dimensions concurrently, we advance beyond a generic understanding of “student diet” towards a more contextualized and actionable view. The following discussion synthesizes key findings in three interconnected areas aligned with our aims: (i) the primary drivers and barriers of canteen service utilization; (ii) the significant disparities in food consumption between the canteen and external contexts; and (iii) the category-specific motivational profiles and the pivotal role of dietary identity in governing food choice. From an applied perspective, this approach is consistent with growing evidence showing that theory-based and data-driven interventions—particularly when tailored to individual and contextual characteristics—are more effective than generic strategies in promoting health-related behaviors [35].

While a substantial body of research has examined university food environments, canteen-based interventions, and individual determinants of students’ dietary behaviors, these components have most often been addressed in isolation [5,7,9,12,18,20,25]. Existing studies have typically focused either on the availability and nutritional quality of campus food offerings, or on individual-level factors such as dietary habits and attitudes, without directly comparing food consumption inside and outside the university setting within a single analytical framework [10,30,31]. As a result, integrated evidence simultaneously linking contextual food environments, individual dietary identity, service engagement, and category-specific decision-making processes remains limited. The present study addresses this gap by combining these levels of analysis to capture how institutional contexts shape the expression of students’ dietary preferences.

### 4.1. Drivers and Barriers of Canteen Engagement: A Service-Centric Perspective

Because institutional food environments play a central role in shaping students’ dietary opportunities, understanding the factors that drive or hinder engagement with the university canteen represents a necessary starting point for interpreting subsequent food choice patterns [9,10]. Moving beyond a simple distinction between users and non-users, examining the motivations underlying canteen use and avoidance provides insight into the contextual constraints and facilitators that condition students’ exposure to institutional food offerings [23,36].

The effectiveness of any canteen-based nutritional intervention fundamentally depends on students’ willingness to use the service. In this regard, our findings clarify the decisive factors in this choice. Cost emerged as the primary driver, consistent with evidence identifying financial constraints as a central concern among university students [20,22,37]. This underscores the canteen’s perceived role as an economically rational choice within the campus foodscape, particularly in a subsidized system where meals are offered at lower prices than those available in surrounding commercial food environments, representing a critical advantage for a student population often managing limited budgets [38].

Conversely, food quality and service time/waiting times were ranked as the top deterrents for non-use. This pattern indicates a critical perception gap: while affordability attracts users, perceived compromises in quality and convenience actively repel a substantial portion of the student body (24.3% were non-users). Such findings align with food service satisfaction models emphasizing the importance of balancing cost with acceptable quality and efficiency [21]. Taken together, these results suggest that interventions aimed at increasing canteen patronage should primarily target core service attributes—such as food quality and waiting times—to broaden students’ exposure to healthier food options. Notably, these criteria were remarkably stable across frequent and infrequent users, pointing to a shared evaluative framework for the canteen service.

This represents a crucial step because, given the established effectiveness of canteen-based interventions for improving students’ dietary choices and health, a primary objective is to increase the number of students who use the university canteen. Identifying the factors that encourage or deter canteen use provides an evidence-based foundation for interventions targeting these factors and, ultimately, for expanding students’ engagement with improved institutional food services.

### 4.2. The Canteen as a Distinct Food Environment: A Pronounced Contextual Consumption Gap

A central and striking finding of this study is the consistent and significant gap in consumption frequency for all six food categories when compared between the canteen and external settings. Consumption was markedly lower within the canteen, with effect sizes ranging from small (e.g., animal-based proteins, d = 0.27) to very large (fruit, d = 0.94). This pronounced “contextual gap” is particularly noteworthy as it applies equally to less healthy items (desserts) and, crucially, to healthier items like fruit and plant-based proteins, which students report consuming more frequently outside. At the same time, this comparison should be interpreted in light of the fact that university canteen services are used almost exclusively for lunch—only rarely for dinner—whereas food consumption outside the canteen typically occurs across multiple meals throughout the day, offering more frequent opportunities for consumption.

This pattern strongly suggests that the current canteen environment may not effectively support or stimulate healthier dietary behaviors, even among students who generally incorporate these foods into their diets elsewhere. Several non-mutually exclusive explanations arise from the literature on food environments [9,10]. First, the limited variety, presentation, or perceived appeal of these items in the canteen may reduce their selection. For instance, the low mean frequency of whole-grain bread (M = 2.11) and fruit (M = 2.11) in the canteen, compared to outside (M = 2.53 and M = 3.69, respectively), points to a failure in the canteen’s “choice architecture” to make these options salient and attractive [15,16]. Second, the canteen may operate as a “default” or habit-driven context where choices are made quickly and automatically, with less active consideration for personal health preferences that might guide more deliberate choices elsewhere [15,16]. Third, practical constraints such as time pressure during lunch breaks may lead students to opt for the most convenient or familiar option, which may not align with their broader dietary patterns [19]. This substantial gap highlights a major opportunity: merely offering healthier foods is insufficient. The canteen environment must be strategically redesigned to bridge this intention–behaviour gap by making healthier choices the easier, more appealing, and more normative option through improved nudges and default [14,18]. Taken together, these findings suggest that the canteen context may attenuate the expression of individual dietary preferences by constraining choice autonomy, thereby amplifying the role of structural and service-related factors.

Beyond identifying the factors that influence canteen use, these findings underscore the importance of examining how food consumption patterns shift across contexts, as the university setting appears to systematically modify students’ dietary behaviors relative to their choices outside the university environment.

### 4.3. Beyond Generic Motives: Category-Specific Profiles of Food Choice

Confirming our aims and addressing a key gap in the literature, our analysis reveals that the importance of food-choice motives is not uniform but varies systematically across food categories. This finding challenges the utility of generic models [12,28], for designing targeted interventions and underscores the need for a more granular approach.

For healthier categories (plant-based proteins, whole-grain bread, fruit), healthiness and digestibility were among the most salient factors.

For fruit, healthiness (M = 4.42) rivalled taste (M = 4.47) as the primary concern. This indicates a deliberate, benefit-oriented decision-making process for these items. In contrast, for less healthy categories (desserts, animal-based proteins), taste and gratification dominated. For desserts, gratification (M = 4.38) was nearly as important as taste (M = 4.57), pointing to a strongly hedonic, reward-driven logic. The direct comparison confirmed that healthiness was significantly more important for healthier foods, while gratification was more important for less healthy ones.

Ethical considerations showed meaningful importance only for plant-based proteins (M = 3.25), aligning with research linking dietary identity to moral concerns about food [27], but were minimal for other categories. This has direct implications for intervention design [39]. Promoting fruit or whole-grain bread should leverage health and digestibility messages and ensure their presentation signals freshness and quality. In contrast, strategies for moderating dessert consumption might focus on altering the hedonic landscape (e.g., reducing portion sizes, improving the taste appeal of healthier alternatives) rather than emphasizing health, which is a low-priority motive for that category. This category-specific approach ensures interventions “speak the same language” as the decision-making process already in play and may improve consumer acceptance of healthy eating strategies [40]. These findings further support the need for data-driven and tailored interventions, as accumulating evidence indicates that theory-based and personalized approaches are more effective in promoting health-related behaviors than generic strategies [35]. Although the present study did not directly assess engagement with digital or mHealth interventions, the strong role of dietary identity and category-specific motivations observed in our analyses suggests potential directions for future intervention development. In this sense, digital approaches may provide a flexible framework for operationalizing the tailoring principles identified in the present findings, rather than representing direct implications of the current data. Such approaches may include tailored dietary feedback aligned with users’ dietary profiles, context-aware prompts delivered around eating occasions, adaptive goal-setting focused on specific food categories, personalized framing of food options emphasizing salient motives such as taste or convenience, and feedback mechanisms that support self-regulation and habit formation over time—an approach that is increasingly well accepted by this population [41,42,43].

An important consideration emerging from the regression analyses concerns the markedly different levels of explained variance across food categories. While models predicting protein-based choices showed moderate to substantial explanatory power (R^2^ = 0.29–0.35 for animal-based proteins and R^2^ = 0.12–0.14 for plant-based proteins), the variance explained for other categories was extremely low. In particular, models predicting fruit consumption explained less than 1% of the variance both inside (R^2^ = 0.008) and outside the canteen (R^2^ = 0.009), while similarly low values were observed for bread products (R^2^ = 0.03–0.05) and desserts (R^2^ = 0.02–0.04). These findings indicate that individual-level predictors such as dietary style, age, and gender account for only a small proportion of variability in the consumption of these foods. Rather than undermining the relevance of the models, this pattern suggests that consumption of fruit, bread, and desserts may be more strongly shaped by contextual and situational factors not directly measured in the present study, such as availability, placement within the serving line, perceived freshness and quality, pricing cues, time constraints during lunch breaks, or momentary hunger [10,30,31]. In this sense, the very low explained variance further supports the interpretation of the university canteen as a food environment in which structural and service-related features may attenuate the expression of individual dietary preferences.

### 4.4. The Pivotal Role of Dietary Identity

Our regression analyses consistently identified self-identified dietary style as the strongest and most stable individual-level predictor of consumption, particularly for protein sources, both inside and outside the canteen. This robust association validates the concept of dietary identity as a powerful cognitive schema that filters food choices across contexts [26,27]. For instance, a stronger orientation towards plant-based diets (e.g., flexitarian, vegetarian, vegan) was the single strongest predictor of lower animal-based protein and higher plant-based protein consumption, with large, standardized coefficients (β = −0.50 and 0.34 in-canteen, respectively).

This finding has profound implications for segmentation and communication strategies. It suggests that canteen interventions, particularly those promoting a shift towards plant-based diets, should move beyond one-size-fits-all messaging. Communications and nudges could be tailored: for omnivores, the focus might be on introducing appealing plant-based options as a tasty “sometimes” choice; for flexitarians, messages could reinforce the positive identity of reducing meat; for vegetarians/vegans, ensuring consistent variety and quality is key. Recognizing these identities allows for more resonant and effective behavioural strategies.

### 4.5. Towards Effective, Tailored Interventions in University Settings

The findings of this study converge on a core principle of contemporary behavioral science: interventions are most effective when they are tailored to both the target population and the specific decision-making context in which behaviors occur [39]. By integrating individual dietary identity, category-specific motivational drivers, and service-level engagement factors, the present study provides a coherent, evidence-based framework for designing such tailored interventions within university canteen settings.

First, the pronounced differences in motivational profiles across food categories underscore the limitations of generic nutritional messaging. Consistent with prior research showing that food choices are guided by distinct combinations of health, sensory, and contextual considerations [28,29], our results indicate that healthier options (e.g., fruit, whole-grain bread, plant-based dishes) are primarily driven by perceived healthiness, digestibility, and taste, whereas less healthy foods are more strongly associated with gratification and hedonic value. This pattern suggests that effective interventions should adopt category-specific strategies rather than uniform approaches across foods.

Second, the substantial gap observed between students’ general dietary habits and their canteen food choices highlights the canteen as a distinct food environment characterized by unique structural and experiential constraints. Previous studies have emphasized how food environments shape behavior through availability, convenience, and perceived quality [9,10]. In line with this evidence, our findings suggest that interventions in university canteens should prioritize environmental re-engineering—such as improving service efficiency, food presentation, and perceived quality—to address barriers that are specific to this context [18,36].

Third, the strong and consistent role of dietary identity as a predictor of food consumption offers a meaningful axis for intervention tailoring and population segmentation. Prior work has shown that self-identity plays a central role in shaping food-related intentions and behaviors, particularly among young adults [8,26]. Leveraging dietary identity (e.g., omnivore, flexitarian, vegetarian) allows interventions to move beyond one-size-fits-all solutions and toward communication strategies that resonate with individuals’ self-concepts and values. This approach aligns with growing evidence that theory-based and tailored interventions are more effective than generic strategies, including in digital health contexts [35,40].

Importantly, the motivational patterns identified in this study point to concrete and testable intervention strategies within the canteen environment. For healthier food categories, interventions that enhance both the actual and perceived sensory quality of foods—such as recipe reformulation, improved presentation, freshness cues, and taste-oriented or descriptive labeling—may be particularly effective. A growing body of literature supports the efficacy of such nudging strategies, including choice architecture modifications, salience enhancement, and default options, in promoting healthier eating behaviors in institutional settings [14,15,16].

Examples of evidence-based interventions include optimizing food placement and visibility, using descriptive naming that emphasizes taste and freshness, adjusting default side dishes toward healthier options, and reducing friction for selecting nutritious foods. Because these strategies act directly on decision-making factors that emerged as salient in our data, they represent theoretically grounded and empirically informed avenues for intervention. More broadly, the observed patterns suggest that students may be receptive to well-designed environmental modifications within the canteen setting, providing a favorable foundation for future, more sophisticated interventions.

### 4.6. Limitations

This study has some limitations that should be considered when interpreting the findings. The cross-sectional design does not allow for causal inferences; future research could address this limitation by adopting longitudinal or experimental designs to examine changes in food choices over time and in response to environmental modifications. The reliance on self-reported measures may have introduced recall and social desirability biases, which could be mitigated in future studies through the integration of objective or momentary assessment approaches, such as food diaries or ecological momentary techniques.

Although the sample size was large, data were collected at a single Italian university, and the gender distribution was skewed toward female participants (68.5%). While this distribution reflects patterns commonly observed in Italian university populations, the single-institution design and the observed gender imbalance may limit the generalizability of the findings, particularly in light of evidence that food environments, pricing structures, and student eating behaviors vary substantially across institutions and national contexts [25]. Multi-site and cross-cultural studies would help to test the robustness of the observed patterns. Age was assessed using categorical ranges and treated as an ordinal predictor in the regression analyses. However, because the vast majority of participants (95.1%) fell within the 18–30 age range, variability in this variable was limited, which likely reduced its explanatory power. Accordingly, any statistically significant age effects observed in the present models should be interpreted with caution, as they may not reflect substantively meaningful or developmentally relevant differences. Future research would benefit from collecting continuous age data, allowing participants to report their exact age in years, which would enable more precise modeling of age-related effects and a more sensitive assessment of potential developmental gradients in food choice behaviors. Finally, an additional limitation concerns the use of a self-reported, frequency-based Likert scale to assess food consumption. While this approach is well suited for large samples and commonly adopted in behavioral research, integrating it with more objective indicators, such as calorie intake, grams, or portion sizes assessed over a defined time window, could provide a more comprehensive picture and allow comparisons between perceived and actual consumption [35,41,44].

## 5. Strengths and Future Directions

### 5.1. Strengths

The primary strength of this study lies in its integrated, multi-level design, which simultaneously examines individual dietary identity, category-specific food-choice motivations, and service-level engagement factors within the university canteen. This approach moves beyond a siloed view of eating behavior and provides a student-centered perspective that is particularly informative for designing context-sensitive interventions.

The large and well-characterized sample further strengthens the robustness of the findings. Finally, the category-specific analysis of decision-making motives represents a novel contribution, offering actionable insights that go beyond generic models of food choice.

### 5.2. Future Directions

Future research should build on these findings by testing interventions that directly target the motivational drivers identified in this study. Canteen-based nudging strategies aimed at improving the perceived and actual quality of healthier food options—such as taste, freshness, and sensory appeal—represent a logical next step, as these factors emerged as central to healthier food choices.

Longitudinal or pre–post intervention designs within the same canteen context would allow comparison of food selection patterns before and after targeted changes, using the present results as an empirical baseline. Incorporating objective indicators of food choice (e.g., purchase or transaction data) would further strengthen inference. Extending this work across different university settings would support assessment of generalizability. Finally, future studies may explore complementary, theory-based mHealth tools designed to deliver personalized support aligned with students’ dietary identities and category-specific motivational profiles identified in the present study.

## 6. Conclusions

This study offers an integrated view of university students’ eating behaviors by jointly considering individual dietary identity, food-choice motivations, and the canteen context. The findings show that food choices within the university canteen do not simply mirror students’ general dietary habits but are shaped by context-specific constraints and category-dependent decision-making processes.

Across contexts, dietary identity emerged as a stable correlate of food consumption, while marked differences across food categories highlighted the limits of uniform approaches to promoting healthier eating. Together, these results indicate that interventions in university canteens should move beyond generic nutritional guidance and instead combine context-sensitive choice architecture with strategies aligned to both food categories and students’ dietary orientations.

By framing the canteen as an active food environment rather than a neutral setting, this study provides an empirical foundation for developing more targeted and effective approaches to support healthier eating during a critical life stage.

## Figures and Tables

**Table 1:** Questionnaire Overview.

Section	Domain	Example Variables
Sociodemographic	Background characteristics	Age, gender, university role
Dietary style	Self-identified dietary style	Omnivorous, pescatarian, flexitarian, vegetarian, vegan
Canteen use	Service utilization	Frequency of canteen use
Food choice motives (canteen)	Decision-making factors	Health, taste, cost, habit, ethics
Food consumption (canteen)	Category-specific intake	Animal-based proteins, plant-based proteins, desserts, fruit
Food consumption (outside)	General dietary behavior	Same categories outside canteen

**Table 2:** Sample Characteristics.

Variable	Category	Frequency	Valid Percentage
Age	18–30	1445	95.1
	31–45	52	3.4
	46–60	20	1.3
	>60	2	0.1
Gender	Male	439	28.9
	Female	1040	68.5
	Non-binary/Third gender	22	1.4
	Prefer not to say	18	1.2
Dietary style	Omnivore	1175	77.4
	Flexitarian	161	10.6
	Pescatarian	31	2.0
	Vegetarian	86	5.7
	Vegan	47	3.1
	Other	19	1.3
Canteen use	No	359	24.3
	Yes	1117	75.7

**Table 3:** Frequency of University Canteen Use.

Frequency of Canteen Use	Percentage (%)
Almost never	22.8
Rarely	14.5
Sometimes	17.3
Often	19.9
Almost always	25.4

**Table 4:** Ranked Reasons for Using the University Canteen (Borda Scores).

Factor	Borda Mean	Total Points	Weight (%)
Cost	4.6948	5000	22.35
Food quality	4.0723	4337	19.39
Distance from usual locations	3.9643	4222	18.88
Service time/waiting time	3.4901	3717	16.62
Presence of friends/colleagues	2.9568	3149	14.08
Habit	1.8216	1940	8.67

**Table 5:** Ranked Reasons for Not Using the University Canteen (Borda Scores).

Factor	Borda Mean	Total Points	Weight (%)
Food quality	3.3661	4561	23.82
Service time/waiting time	3.4354	4655	24.06
Cost	2.8863	3911	20.43
Canteen environment	2.5683	3480	18.13
Distance from usual locations	2.7439	3718	19.56

**Table 6:** Consumption of Selected Food Categories in the University Canteen.

Food Category	Valid N	Mean (M)	Standard Deviation (SD)
Animal-based proteins	1030	3.49	1.19
Plant-based proteins	994	3.15	1.16
White bread	962	2.85	1.48
Whole-grain bread	930	2.11	1.28
Desserts	915	2.27	1.38
Fruit	915	2.11	1.3

**Table 7:** Consumption of Selected Food Categories Outside the University Canteen.

Food Category	Valid N	Mean (M)	Standard Deviation (SD)
Animal-based proteins	1236	3.74	1.01
Plant-based proteins	1236	3.64	0.97
White bread	1236	3.11	1.22
Whole-grain bread	1236	2.53	1.16
Desserts	1236	2.98	1.04
Fruit	1236	3.69	1.14

**Table 8:** Comparison of Consumption of Selected Food Categories inside and outside the University Canteen.

Food Category	Paired N	Mean (Canteen)	Mean (Outside Canteen)	Mean Difference (Canteen–Outside)	*p*-Value	Effect Size (Cohen’s d)
Animal-based proteins	897	3.47	3.74	−0.26	<0.001	0.27
Plant-based proteins	897	3.15	3.64	−0.49	<0.001	0.43
White bread	897	2.85	3.11	−0.26	<0.001	0.16
Whole-grain bread	897	2.11	2.53	−0.42	<0.001	0.34
Desserts	897	2.27	2.98	−0.73	<0.001	0.53
Fruit	897	2.11	3.69	−1.31	<0.001	0.94

Note. Values represent mean consumption frequencies. Mean differences refer to canteen minus outside-canteen consumption. Effect sizes are reported as Cohen’s d. *p*-values refer to paired-samples comparisons. *p* < 0.05; *p* < 0.01; *p* < 0.001.

**Table 9:** Importance of Decision-Making Factors Across Food Categories within the Canteen.

Factor	Animal-Based Proteins (Mean ± SD)	Plant-Based Proteins (Mean ± SD)	White Bread (Mean ± SD)	Whole-Grain Bread (Mean ± SD)	Desserts (Mean ± SD)	Fruit (Mean ± SD)
Taste	4.39 ± 0.71	4.33 ± 0.80	4.07 ± 0.91	4.13 ± 0.85	4.57 ± 0.61	4.47 ± 0.68
Healthiness	3.95 ± 0.96	4.14 ± 0.90	3.31 ± 1.19	3.99 ± 1.02	3.09 ± 1.26	4.42 ± 0.83
Digestibility	3.78 ± 1.09	3.75 ± 1.09	3.38 ± 1.20	3.69 ± 1.11	3.40 ± 1.24	3.83 ± 1.20
Gratification	3.77 ± 0.97	3.72 ± 1.01	3.71 ± 1.05	3.62 ± 1.08	4.38 ± 0.82	3.87 ± 0.99
Cost/Price	3.88 ± 1.14	3.72 ± 1.18	3.37 ± 1.35	3.38 ± 1.36	3.59 ± 1.23	3.47 ± 1.32
Appearance/Presentation	3.57 ± 1.10	3.51 ± 1.16	3.26 ± 1.23	3.30 ± 1.18	3.94 ± 0.99	3.88 ± 1.13
Habit	2.91 ± 1.08	3.06 ± 1.14	3.31 ± 1.20	3.15 ± 1.26	3.04 ± 1.21	3.59 ± 1.21
Ethical considerations	2.93 ± 1.27	3.25 ± 1.35	2.59 ± 1.27	2.97 ± 1.35	2.69 ± 1.28	3.06 ± 1.42

**Table 10:** Comparison of Decision-Making Factors for Healthier vs. Less Healthy Foods in the University Canteen.

Factor	Mean_Healthy	Mean_Unhealthy	Difference_H_Minus_U	*t*_Value	*p*_Value_Two_Tailed	Cohens_d
Healthiness	4.15	3.6	0.55	19.24	<0.001	0.64
Taste	4.33	4.31	0.02	0.74	0.461	0.03
Digestibility	3.74	3.6	0.13	6.23	<0.001	0.21
Gratification	3.73	3.85	−0.12	−4.75	<0.001	−0.16
Cost	3.61	3.66	−0.05	−2.5	0.012	−0.08
Appearance	3.59	3.55	0.05	2.31	0.021	0.08
Habit	3.21	3.07	0.14	4.99	<0.001	0.17
Ethical considerations	3.1	2.83	0.24	9.93	<0.001	0.33

Note. Values represent mean importance ratings. Mean differences refer to healthier minus less healthy foods. Effect sizes are reported as Cohen’s d. *p*-values refer to paired-samples *t*-tests. *p* < 0.05; *p* < 0.01; *p* < 0.001.

**Table 11:** Standardized Regression Coefficients (β) and Model Fit for Food Consumption withing the Canteen.

Food Category	Dietary Style (β)	Age (β)	Gender (β)	Model R^2^
Animal-based proteins	−0.50 ***	−0.07 **	−0.11 ***	0.293
Plant-based proteins	0.34 ***	n.s.	n.s.	0.122
White bread	−0.11 ***	−0.11 ***	−0.13 ***	0.045
Whole-grain bread	0.14 ***	0.11 **	n.s.	0.033
Desserts	−0.16 ***	−0.10 **	n.s.	0.040
Fruit	0.09 **	n.s.	n.s.	0.008

Note. Values represent standardized regression coefficients (β). n.s. = not significant. ** *p* < 0.01; *** *p* < 0.001.

**Table 12:** Standardized Regression Coefficients (β) and Model Fit for Food Consumption outside the Canteen.

Food Category	Dietary Style (β)	Age (β)	Gender (β)	Model R^2^
Animal-based proteins	−0.57 ***	−0.12 ***	−0.14 ***	0.353
Plant-based proteins	0.36 ***	n.s.	0.12 **	0.138
White bread	−0.11 ***	−0.14 ***	−0.13 ***	0.033
Whole-grain bread	0.16 ***	n.s.	n.s.	0.032
Desserts	n.s.	−0.11 ***	0.09 **	0.016
Fruit	0.08 **	n.s.	n.s.	0.009

Note. Values represent standardized regression coefficients (β). n.s. = not significant. ** *p* < 0.01; *** *p* < 0.001.

## Data Availability

The data presented in this study are available from the corresponding author upon reasonable request. The data are not publicly available due to privacy and ethical restrictions.
